# Does Gender Inequity Increase the Risk of Intimate Partner Violence among Women? Evidence from a National Bangladeshi Sample

**DOI:** 10.1371/journal.pone.0082423

**Published:** 2013-12-23

**Authors:** Mosiur Rahman, Keiko Nakamura, Kaoruko Seino, Masashi Kizuki

**Affiliations:** 1 International Health Section, Division of Public Health, Graduate School of Tokyo Medical and Dental University, Tokyo, Japan; 2 Health Promotion, Division of Public Health, Graduate School of Tokyo Medical and Dental University, Tokyo, Japan; Indiana University, United States of America

## Abstract

**Background:**

Evidence from developing countries regarding the association between gender inequity and intimate partner violence (IPV) victimization in women has been suggestive but inconclusive. Using nationally representative population-based data from Bangladesh, we examined the association between multidimensional aspects of gender inequity and the risk of IPV.

**Methods:**

We used data from the 2007 Bangladesh Demographic Health Survey. The analyses were based on the responses of 4,467 married women. The main explanatory variable was gender inequity, which reflects the multidimensional aspects of women's autonomy and the relationship inequality between women and their partner. The experience of physical and/or sexual IPV was the main outcome variable of interest.

**Results:**

Over 53% of married Bangladeshi women experienced physical and/or sexual violence from their husbands. In the adjusted models, women who had a higher level of autonomy (adjusted odds ratio [AOR] 0.48; 99% confidence interval [CI] 0.37–0.61), a particularly high level of economic-decision-making autonomy (AOR 0.12; 99% CI 0.08–0.17), and a higher level of non-supportive attitudes towards wife beating or raping (AOR 0.61; 99% CI 0.47–0.83) were less likely to report having experienced IPV. Education level, age at marriage, and occupational discrepancy between spouses were also found to be significant predictors of IPV.

**Conclusions:**

In conclusion, dimensions of gender inequities were significant predictors of IPV among married women in Bangladesh. An investigation of the causal link between multidimensional aspects of gender inequity and IPV will be critical to developing interventions to reduce the risk of IPV and should be considered a public health research priority.

## Introduction

Intimate partner violence (IPV), which consists of a range of physical or sexual coercive acts, or both, perpetrated against women by a current or former male intimate [1], has emerged as a significant public health concern around the world. Awareness of the wide range of mental, physical, reproductive, and sexual health consequences of IPV has been increasing [2]–[6]. Recent international estimates indicate that the percentage of women with a lifetime experience of IPV is as high as 71% (rural Ethiopia) [7] and falls between 21% and 47% in the majority of countries [8]. IPV is acute in South Asia, a region where issues related to basic gender equity have been much discussed [9]–[11]. In Bangladesh, which accounts for 9.2% of the South Asian female population, some 69% of women have reported being physically and/or sexually abused by their husbands during their life time [8].

To date, the major determinants that influence the risk of IPV among women in poorer settings are assumed to be related to inadequate financial resources, low levels of household wealth [12], larger family sizes, more children under the age of five years [13], and certain general community and lifestyle related factors [14]–[15]. However, other socio-cultural determinants of IPV, such as gender inequity, are not yet well understood. Gender inequity, which is influenced by historical and structural power imbalances between women and men, can increase the risk of acts of violence by men against women, For instance, traditional beliefs that men have a right to control women make women vulnerable to violence by men [8]. They also hinder the ability of those affected to remove themselves from abusive situations or to seek support [16]. Understanding the association between gender inequities and IPV is therefore vital for the development of effective IPV prevention programs.

In highly patriarchal societies, such as Bangladesh, where traditional gender paradigms exist, women, in both custom and practice, have remained subordinate to men in almost all aspects of their lives. A woman's freedom to exercise her own judgment and to act in her own interests is greatly restricted [17], [18]. The family is the central focus, and women are identified as mothers and wives, rather than as individuals in their own right. The ongoing perception is of men's responsibility to be the sole breadwinner and the placement of restrictions on the behavior and mobility of women [19]. These inequalities can increase the risks of abuse, violent relationships and exploitation of women.

A limited number of studies from South Asia [20]–[22] and from diverse international settings [23]–[25] outside South Asia have shown a link between IPV and gender inequity. However, evidence from these studies remains inconclusive. For example, studies from India and Nepal have found that control over financial resources was associated with a reduced risk of IPV [20]–[21]. In contrast, studies from Haiti, Peru, Bangladesh, and Brazil found that higher autonomy, as measured by financial autonomy, was associated with elevated risks of violence from intimate partners [22]–[25]. In addition, a multi-country analysis showed that in Bolivia, Haiti, and Malawi, women who made decisions regarding their own health care independently were more likely to experience violence from their husband than those who made decisions jointly together with their husband [26]. Multiple elements may possibly account for such variability, such as context diversity or differences in the available measures for gender inequity.

Moreover, methodological issues related to the measurement of gender inequity have not been adequately addressed in previous studies. The complexity of measuring gender inequity lies in its multidimensional nature. The inequalities between men and women are manifested in a variety of dimensions, and are present in different dimensions for different contexts and individuals. Most studies in this area have mainly focused on economic empowerment and some household decision-making variables, and have not depicted the multidimensional aspects of gender inequity and their effects on IPV. Additionally, we contend that the multidimensionality of women's autonomy-the fact that a woman might be highly empowered in one aspect, such as freedom of movement, but poorly empowered in another, such as economic-decision-making power- is a factor that contributes to the complexity of studies examining the links between this process and the risk of IPV. Therefore, a more empirical analysis is needed to enable a clear understanding of the association between different dimensions of control in relationships, which may lead to progress in understanding the risk of IPV. Based on these considerations and using nationally representative population based data from Bangladesh, we therefore aimed to examine the relative importance of gender inequity on the risk of IPV by distinguishing four different dimensions of women's autonomy (women's economic-decision-making autonomy, familial health care and family planning decision-making autonomy, extent of freedom of movement autonomy, and women's attitudes toward partner's violence) and three different dimensions of relationship inequality (educational, age at marriage, and occupational discrepancy between spouses).

## Methods

### Data Sources

The current cross-sectional study used data from the Bangladesh 2007 Demographic and Health Survey (2007 BDHS) conducted under the authority of the National Institute for Population Research and Training (NIPORT) of the Ministry of Health and Family. A stratified, multi-stage cluster sample of 361 primary sampling units was constructed (134 in urban areas and 227 in rural areas). The primary sampling units were derived from a sampling frame created for the 2001 Bangladeshi census. The BDHS uses extensive interviewer training, standardized measurement tools and techniques, an identical core questionnaire, and instrument pretesting to ensure standardization and comparability across diverse sites and time [27].

Trained data collectors performed face-to-face interviews with an adult member in each of 10,819 selected households to obtain demographic information about the household and family members, achieving a household response rate of 99%. The high response rate for the BDHS was attributed to the rigorous training of field staff and close supervision of the fieldwork. Moreover, numerous efforts were made during fieldwork to ensure high response rates: interviewers were trained to maintain motivation with longer questionnaires, probe for responses, clarify ambiguous questions, perform multiple revisits to the household, and to control the order of the questions. Field work was monitored through visits by representatives from U.S. Agency for International Development (USAID), MEASURE DHS, and NIPORT using additional quality control teams. In addition, to achieve the target number of sample units, non-response rates for sample units were estimated from past surveys at the time of the sample design and were then used to determine the required number of units to be selected [27].

**Figure 1:**
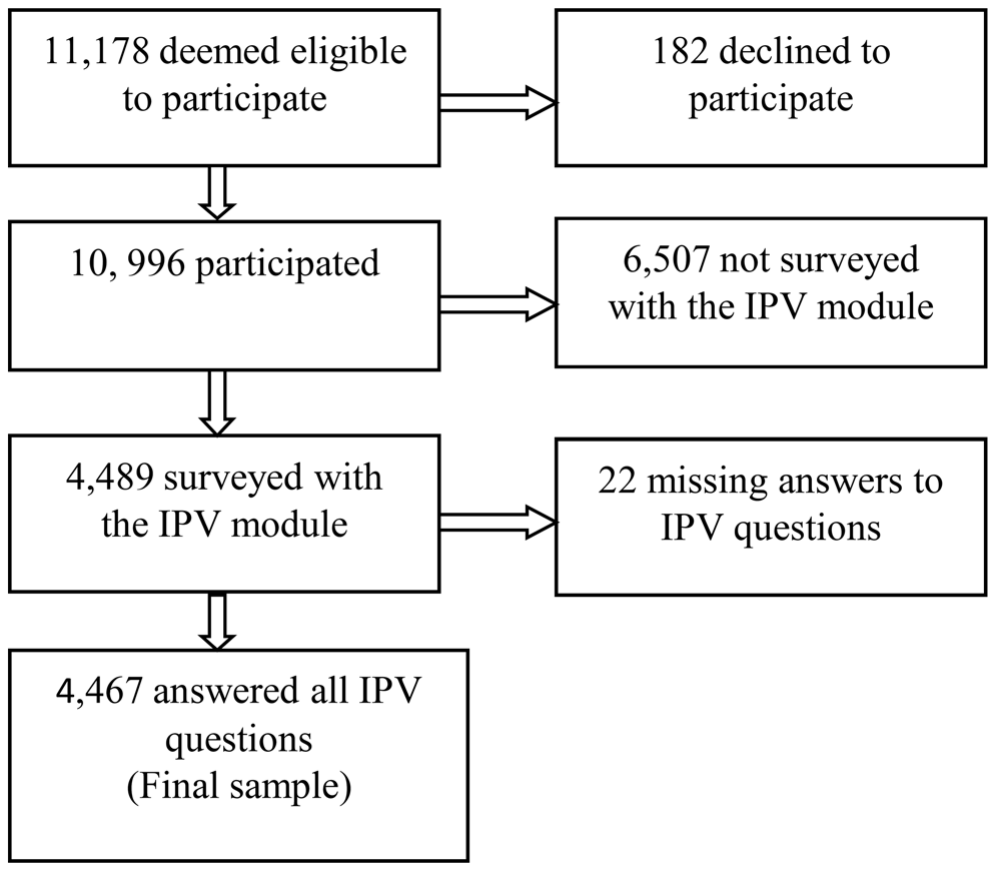
Selection of the sample. From the original 11,178 eligible women, we obtained a final sample of 4, 467 ever married women for this study, 2007 Bangladesh Demographic Health Survey.

The 2007 BDHS used five questionnaires. The questionnaires were drafted in English and were then translated into Bangla, the national language of Bangladesh. The translations were reviewed by experts and volunteers, and a pilot study was conducted as a validation exercise. Of the 11,178 eligible women in the households that were surveyed, 98.4% participated in a survey of maternal and child health behaviors and outcomes. The domestic violence module was a relatively new addition to BDHS and was administered to one, randomly selected, woman per household, who was asked to answer an additional set of questions regarding IPV perpetrated by her husband. This module yielded complete data from 4,467 married women (Figure 1).

### Intimate Partner Violence

After consultation with experts, the 2007 BDHS used a standardized approach to measure IPV. This approach involved implementing the most valid measure available: a shortened and modified version of the Conflict Tactics Scale (CTS-2). The 2007 BDHS stated that to ensure the validity and reliability of data collected regarding IPV, fieldworkers underwent careful training in different aspects of interview techniques and the questionnaires were pre-tested in pilot studies [27]. Moreover, to make valid cross-national comparisons, the questionnaire that was used to measure IPV in the BDHS included the same criteria and methods used for all cultural contexts [27].

The perpetration of IPV by the woman's husband was assessed via 8 survey items. Women who reported that their husband engaged in any of the following behaviors were classified as having experienced physical and/or sexual IPV: 1) pushing, shaking, or throwing an object; 2) slapping; 3) pulling hair or twisting an arm; 4) punching or hitting with a fist or something harmful; 5) kicking or dragging; 6) choking or burning; 7) threatening or attacking with a knife or gun; or 8) physically forcing her to have sexual intercourse with him even when she did not want to. The recall period was defined as ‘since the age of 15 years’.

### Explanatory Variables

The main explanatory variable in our study was gender inequity, which reflects the multi-dimensional aspects of women's autonomy and relationship inequality between spouses.

#### Women's Autonomy Indicators

The selection of autonomy variables was determined based on two criteria: (1) they had been measured in the BDHS data, and (2) existing literature [28]–[32] had used them to represent autonomy. We began with a set of 11 items thought to reflect autonomy. The degree of women's autonomy was assessed in four different areas: women's economic-decision-making autonomy, familial health care and family planning decision-making autonomy, extent of freedom of movement autonomy, and women's attitudes toward partner's violence. Women's economic dependency has long been understood to be a major factor in structuring inequalities between men and women [33]. Hence, control over financial resource is often considered to be a central dimension when measuring women's autonomy. The more control a woman has over her own time and household income, the greater the likelihood that she may be able to increase her capacity to challenge the acceptability of partner violence. Thus, we hypothesized that a woman's economic independence was likely to be an important determinant of IPV. To measure the economic-decisions-making autonomy of women the BDHS survey asked women three questions related to control over earnings: “Who in your family has the final say on: 1) how the money you earn will be used?; 2) making large household purchases?; and 3) making household purchases for daily needs?”

To measure aspects of familial health care and family planning decision-making autonomy, the survey asked the following two questions: 1) “Who in your family makes decisions about healthcare for yourself?” and 2) “Does the respondent discuss family planning with her partner?” Discussion of this topic is obviously important, because evidence shows that victims of IPV have a lower ability to make decisions regarding the choice to receive appropriate health care [5]. Furthermore, evidence suggests that women who experience IPV have reduced access to family planning or other fertility control resources [7], [21]. Hence, we considered familial health care and family planning decision-making to be an important dimension of women's autonomy in discussions of IPV.

Freedom of movement is also crucial for participation in daily economic and social activities and is therefore vital for being actively in charge of one's' life. To measure the extent of freedom of movement autonomy, the following two questions were asked: “Who in your family has the final say on: 1) visits to family or relatives? and 2) going to a health center or hospital?” Access to these two locations is vital for both a woman's well-being and the shaping of her immediate environment. For example, women whose movements are restricted and whose interactions with relatives or friends are closely monitored by their husband or in–laws are expected to have a lack of kin and non-kin social and medical support and fewer resources to protect them from IPV [34].

For each of the questions, the responses were coded as: 1) respondent, 2) respondent and husband/partner jointly, 3) respondent and someone else, 4) husband/partner, or 5) someone else in the household. To assess the respondent's autonomy, binary variables were created for each of the questions by merging responses 1, 2, and 3 into one category representing decision-making power and merging responses 4 and 5 into one category representing no decision-making power. Only those women who had paying jobs were asked the question about who decided how the money that they earned would be used. Women who did not have jobs were considered not to have decision-making power.

In addition, an index of gender-roles ideology was constructed based on the women's answers to four questions regarding the correct attitude that a woman should have in a particular situation: 1) if she goes out without informing her husband; 2) if she argues with her husband; 3) if she neglects the children; and 4) if she refuses to have sex with her husband. For each of these questions, responses were coded as yes (1) or no (2). These were general attitude questions, rather than questions asking the women about their own experiences. The assumption with these questions is that women with decision-making power would not accept such obvious gender inequalities in power and would not agree with any justification for a husband beating or raping his wife, and this assumption is in agreement with findings from other studies [35]–[36]. Moreover, an additional analysis was performed in our study to support this hypothesis, and women with high economic decision-making autonomy were found to have a low agreement with partner violence justification. We defined a variable with two categories from these questions to separate respondents who felt that wife beating or raping was not justifiable for any reason and respondents who felt that wife beating or raping was justifiable for any single or several reasons.

#### Relationship Inequality

Relationship inequality in this study was assessed based on education level, age at marriage, and occupational discrepancy between spouses via responses to the BDHS questionnaire given to women. The available literature suggests that husbands with a higher educational status than their wives are more likely to assert unequal and even violent power in their relationship [37]. Various other studies have also suggested that when wives have a greater educational status than their husbands, there is an increased risk of marital discord [37]–[38]. Therefore, a variable was created to define spousal educational inequalities in the following manner. First, a binary variable was created to assess the level of the woman's education, as either illiterate or literate. Second, we considered the educational level of the spouse in the same manner. Third, we analyzed the educational discrepancy between spouses according to 4 categories as follows: 1) both the respondent and her husband were literate; 2) only the husband was literate; 3) only the respondent was literate; and 4) both the respondent and her husband were illiterate. When women have a higher employment status than their spouse, it may lead to a sense of powerlessness in men and because of their social position, they may feel ‘unsuccessful’ as men, further increasing the risk of IPV [39]. Therefore, a variable for occupational inequalities between spouses was created and was categorized as follows: 1) only the husband had a job; 2) both the respondent and her husband had a job; 3) only the respondent had a job; and 4) both the respondent and her husband did not have jobs.

A variable was created to define discrepancy in age between spouses at the time of marriage in the following manner. First, a binary variable was created to assess whether the woman had married before the age of 18 years. Second, we considered the age of the spouse at the time of marriage in the same manner (whether the spouse married before the age of 21 years). We considered the legal age for marriage to be 21 years for men and 18 years for women according to the National Child Marriage Restraint Act in Bangladesh. Third, we analyzed the discrepancy in age between spouses at the time of marriage according to 3 categories as follows: 1) both husband and wife married at legal age; 2) husband or wife married at illegal age; and 3) both husband and wife married at illegal age.

### Covariates

This study included several socioeconomic and demographic variables that have been theoretically and empirically linked to IPV [12]–[13], [15], [40]. The participants' age were categorized as follows: 15–24, 25–34, and 35–49 years of age. Age was included here because several anthropological and empirical studies undertaken in disparate cultures have found that age is negatively associated with the experience of IPV [41], [42]. Support for the husband's education level as a risk factor for IPV varies [15], so, a variable for the husband's education level was created and was categorized as illiterate versus literate. Tertiles were used to classify the total number of household members (1–4, 5–6, or 7 or more). The place of residence was categorized as rural versus urban. Religion was categorized as Muslim versus non-Muslim. High fertility has been associated with IPV, both as a potentially causal factor [43] as well as an outcome of IPV [44], so a variable for parity was created and was categorized as no children or 1, 2, or 3 or more children. We classified the frequency of mass media exposure, which was found to be a strong predictor of IPV in developing countries [45], into three categories: regularly, irregularly, or not at all. Tertiles were used to classify the duration of marriage (<10 years, 10–19 years, or 20 years or more). The BDHS wealth index was used as a proxy indicator of socioeconomic position. The wealth index was constructed from data on household assets, including ownership of durable goods (such as televisions and bicycles) and dwelling characteristics (such as source of drinking water, sanitation facilities and construction materials). Principal components analyses were used to assign individual household wealth scores. These weighted values were then summed and rescaled to range from 0 to 1, and each household was assigned to the poorest, middle or richest tertile [27].

### Statistical Analyses

The prevalence of lifetime physical and/or sexual IPV was estimated for the total sample of married Bangladeshi women and according to demographics. Demographic differences in IPV perpetration were assessed using χ2 analyses. To account for the problem of multiple hypotheses testing, we set the threshold for significance to *P* = 0.05/8 = 0.00625 by using Bonferroni Correction. This was because we tested 8 hypotheses. This conservative correction is used to correct for biased tests in sets of hypotheses leading too many results showing as significant [46]. Analyses were performed using Stata version 11.0 (Stata Corp., College Station, TX, USA). ‘Svy’ commands were used to allow for adjustments for the cluster sampling design, sampling weights, and the calculation of standard errors. These commands use a Taylor series linearization method to estimate confidence intervals around prevalence estimates. Logistic regression models were fitted to the data to model the crude associations between dimensions of gender inequities and IPV. Socio-demographic explanatory variables were then added into the models. This was done to see how the addition of other variables affected the relationship between dimensions of gender inequities and IPV. We created three fully multivariate logistic regression models to analyze the occurrence of IPV among women, with each model containing a different gender inequity predictor.

We entered all the covariates simultaneously into the multiple regression models. Multi-colinearity in the logistic regression analyses was checked by examining the standard errors of the regression coefficients. A standard error larger than 2.0 indicates numerical problems such as multi-colinearity, among the independent variables [47]. However, in our study, all the independent variables in all the adjusted models had a standard error <0.30, indicating an absence of multi-colinearity. We estimated the odds ratios (ORs) to assess the strength of the associations and used the 99% confidence intervals (CIs) for significance testing.

The indices for each of the four dimensions of autonomy, namely the economic-decision-making index (EDI), the index of familial health and family planning decision-making autonomy (FFI), the index of freedom of movement (FMI), and the index of women's attitudes toward partner violence (WAVI), were constructed using the sums of weighted binary input variables, where the maximum and minimum values were chosen for each underlying indicator. The performance of each indicator was expressed using a unit-free index between 0 and 1 (which allows the different indices to be added together) in accordance with the construction method of the Human Development Index [48] as follows:
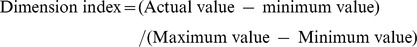
The scores obtained for each of the indices were then recoded as tertiles with the categories labeled as low, middle, and high autonomy. The overall women's autonomy index (OAI) was then computed by averaging the factor scores of these four indices recoded as follows:
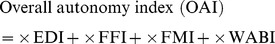
The scores obtained were then recoded as tertiles with the categories labeled as low, middle, and high autonomy. We used Cronbach's alpha coefficients to assess the internal reliability of the overall autonomy index. Cronbach's alpha for these 11 sub-questions was 0.75, suggesting a high internal consistency.

### Ethical Considerations

The data collection procedures for the BDHS were approved by the ORC Macro-institutional review board. The survey protocol was reviewed and approved by the National Ethics Review Committee of the Bangladesh Ministry of Health and Family Welfare. Because the existence of a signed consent form can itself present a risk for abused women, oral informed consent was obtained from the respondents by the interviewers. The original survey was administered in accordance with the World Health Organization (WHO)'s ethical and safety guidelines for research on IPV. These included attaining individual informed consent and ensuring privacy and confidentiality so as to protect the safety of both the respondents and the field staff [27]. If any of the respondents had ever experienced IPV and sought assistance (requiring either medical or psychological help), then the interviewers provided a list of organizations that assist women in distress. This list was provided to the interviewers, and the interviewers were specially trained to provide this information in a confidential and safe manner if requested by the respondents [27].

In addition, to protect the participants from coercion, the following steps were taken by the BDHS: at the beginning of the interview, respondents were read a document emphasizing the voluntary nature of the survey, outlining the potential risks, and explaining that the information gathered would be used to assess health needs and to plan health services; an additional statement was provided to inform them that the questions that would be asked could be of a sensitive nature and to reassure them of the confidentiality of their responses; if privacy could not be ensured, the interviewer was instructed to skip the module. The option of discontinuing the interview if complete privacy could not be obtained increases the likelihood that violence questions are asked only when the respondent feels secure. Interviewers were all provided with a list of organizations helping women in difficult situations. This study was considered to be exempt from a full review as it was based on an anonymous public use of a secondary data set with no identifiable information regarding the survey participants.

## Results

### Descriptive Statistics

Approximately one-third of the respondents (32.9%) were 15–24 years old, 35% had no education, 91% were Muslim, and 77% lived in rural areas (Table 1). Regarding their occupation status, 67.8% of the women had no jobs. About 29.4% of the women had a household member size of seven or more, 47.6% had three or more children, and 30.3% watched mass media regularly (watching TV). From the total sample population, 41.3% of the women were defined as being rich, 19.2% belonged to the middle band of wealth, and 39.5% were poor.

**Table 1:** Descriptive statistics, according to IPV experienced by married women: 2007 Bangladesh Demographic Health Survey (n = 4,467).

Characteristic	Number	% (99% CI)	Any physical and/or sexual IPV% (99% CI)
**Age, years**			
15–24	1,313	32.9 (30.6–35.3)	48.3 (43.2–53.3)
25–34	1,610	33.4 (31.6–35.2)	55.6 (51.4–59.8)
35–49	1,544	33.7 (31.4–36.1)	55.7 (51.2–60.1)
*P* Value			**0.002**
**Age at marriage, years**			
<15	2,679	62.9 (59.9–65.7)	58.5 (55.0–61.8)
≥15	1,788	37.1 (34.3–40.1)	44.4 (40.0–48.9)
*P* Value			**<0.001**
**Education**			
Illiterate	1,496	35.0 (31.9–38.0)	61.9 (57.2–66.3)
Literate	2,971	65.0 (62.0–68.1)	48.6 (45.1–52.1)
*P* Value			**<0.001**
**Husband's education**			
Illiterate	1,536	36.3 (33.5–39.3)	61.5 (57.1–65.7)
Literate	2,928	63.6 (60.7–66.4)	48.6 (45.1–52.0)
Data missing	3		
*P* Value			**<0.001**
**Area of residence**			
Rural	2,798	77.0 (75.1–78.5)	54.9 (51.4–58.4)
Urban	1,669	23.0 (21.5–24.9)	47.6 (42.3–52.9)
*P* Value			**0.003**
**Religion**			
Muslim	4,036	91.0 (87.1–93.6)	54.2 (51.1–57.3)
Non-Muslim	430	9.0 (6.4–13.6)	43.4 (36.0–51.2)
Data missing	1		
*P* Value			**<0.001**
**Respondent currently working**			
No	3,090	67.8 (64.5–70.8)	49.2 (45.7–52.7)
Yes	1,376	32.2 (29.2–35.5)	61.8 (57.4–66.0)
Data missing	1		
*P* Value			**<0.001**
**Parity, no. of children**			
0	353	9.8 (8.1–11.7)	34.8 (26.3–44.5)
1	833	19.6 (17.7–21.5)	46.7 (40.0–53.1)
2	1,090	23.0 (20.8–25.5)	54.4 (48.8–59.8)
3+	2,191	47.6 (44.9–50.4)	59.1 (55.1–63.1)
*P* Value			**<0.001**
**Number of household members (tertiles)**			
1–4	1,849	37.0 (34.7–39.4)	55.4 (51.4–58.7)
5–6	1,583	33.6 (31.4–35.9)	55.1(51.4–59.5)
≥7	1,035	29.4 (26.9–31.9)	48.4 (43.0–53.8)
*P* Value			**0.003**
**Marital duration, years**			
<10	1,512	36.1 (33.7–38.5)	46.3 (41.5–51.2)
10–19	1,523	31.8 (29.8–33.9)	56.8 (52.4–61.1)
≥20	1,432	32.1 (29.8–34.4)	57.5 (53.0–61.9)
*P* Value			**<0.001**
**Frequency of mass media exposure**			
Not at all	2,072	46.0 (42.4–49.7)	54.1 (50.3–57.8)
Irregularly	994	23.6 (21.4–26.0)	59.1 (53.7–64.3)
Regularly	1,398	30.3 (27.6–33.2)	47.4 (41.8–53.0)
Data missing	3		
*P* Value			**<0.001**
**Wealth index category**			
Poor	1,662	39.5 (35.9–43.2)	60.3 (56.4–64.2)
Middle	850	19.2 (17.0–21.6)	58.5 (52.3–64.4)
Rich	1,955	41.3 (37.8–44.9)	44.0 (39.6–48.5)
*P* Value			**<0.001**
**Economic power decision index**			
Low	1,371	30.9 (28.5–33.5)	63.5 (58.5–68.2)
Medium	1,376	31.3 (29.0–33.7)	67.0 (62.5–71.2)
High	1,713	37.8 (35.3–40.3)	33.4 (29.1–38.0)
Data missing	7		
*P* Value			**<0.001**
**Familial health care and family planning decision index**			
Low	872	19.8 (17.9–21.9)	54.7 (48.8–60.3)
Medium	2,308	52.2 (49.9–54.6)	54.6 (50.9–58.4)
High	1,287	27.9 (25.7–30.2)	49.6 (44.6–54.6)
*P* Value			0.059
**Index for freedom of movement**			
Low	596	14.5 (12.7–16.5)	47.9 (40.6–55.3)
Medium	1,776	40.1 (37.7–42.6)	51.7 (47.6–55.9)
High	2,091	45.3 (42.6–48.1)	56.3 (52.3–60.0)
Data missing	1		
*P* Value			**0.008**
**Index of women's attitudes toward partner's violence**			
Low	839	17.5 (15.4–19.8)	63.5 (57.9–68.7)
Medium	578	12.3 (10.8–14.0)	57.1 (49.8–64.2)
High	3,060	70.2 (67.3–72.8)	50.0 (46.6–53.4)
*P* Value			**<0.001**
**Overall autonomy index**			
Low	1,738	39.5 (36.8–42.3)	57.8 (53.6–61.8)
Medium	1,217	28.0 (25.8–30.3)	55.5 (50.7–60.2)
High	1,504	32.5 (29.9–35.2)	45.8 (41.4–50.4)
Data missing	8		
*P* Value			**<0.001**
**Educational inequality**			
Both respondent and her partner were literate	2,290	49.3 (46.3–52.4)	44.3 (40.6–48.1)
Only husband literate	549	12.3 (10.7–14.0)	57.1 (49.8–64.1)
Only respondent literate	700	16.2 (14.5–18.1)	63.0 (56.9–68.8)
Both respondent and her husband were illiterate	925	22.2 (19.7–24.8)	63.8 (58.3–68.9)
Data missing	3		
*P* Value			**<0.001**
**Occupational inequality**			
Both respondent and her husband had a job	1,329	31.3 (28.3–34.5)	61.5 (57.1–65.7)
Only husband had a job	2,968	65.0 (61.7–68.1)	49.4 (45.8–53.0)
Only the respondent had a job	42	8.8 (5.2–14.8)	67.9 (37.4–88.2)
Both respondent and husband did not have any job	123	2.8 (2.1–3.9)	48.0 (32.2–64.2)
Data missing	5		
*P* Value			**<0.001**
**Inequality in spousal age at marriage** †			
Both husband and wife married at legal age	792	15.3 (13.3–17.6)	36.8 (30.9–43.2)
Husband or wife married at illegal age	2,472	54.9 (52.1–57.7)	53.0 (49.1–56.8)
Both husband and wife married at illegal age	1,203	29.8 (27.1–32.5)	62.2 (57.2–66.9)
*P* Value			**<0.001**
**Prevalence**			**53.2**

† Legal marriage is 21 years for men and 18 years for women.

From the total sample population, 32.5% of the women had high overall autonomy: 37.8% had high economic-decision-making autonomy, 27.9% had high familial health care and family planning decision-making autonomy, 45.3% had high freedom of movement autonomy, and 70.2% of women had high levels of non-supportive attitudes toward wife beating or raping (Table 1). Approximately half of the participants and their husbands were both literate, while 65% of the respondent's husbands were employed. A substantial percentage of women (53.2%) reported that they had suffered physical and/or sexual IPV at some point in their life (Table 1).

The bivariate analyses revealed several significant differences in the prevalence of IPV perpetration across various socio-demographic groups (Table 1). Specifically, younger women (those aged 15–24 years) were significantly less likely to report physical and/or sexual IPV than older women. In addition, illiterate women and women who lived in rural areas were significantly more likely to report any form of IPV than literate women and those who lived in urban areas, respectively.

Reports of any form of IPV were significantly more frequent among women who were employed and women belonging to households afflicted by poverty. Respondents having three or more children and who watched mass media irregularly (watched TV) had a higher risk of IPV than their counterparts (Table 1). A higher prevalence of any form of IPV was identified only among Muslim women and women living in smaller households (1–4 persons) (Table 1).

Respondents, who had a high level of economic-decision-making autonomy, a low level of freedom of movement autonomy, a high level of women's attitudes toward partner's violence autonomy, and a high overall level of autonomy were less likely to report any form of IPV (Table 2). Reports of any form of IPV were significantly more frequent among women, who reported that both they and their husbands were illiterate. A higher prevalence of IPV was observed when only the respondents were employed. In addition, a higher prevalence of IPV was observed among respondents when both of the spouses married at an illegal age (Table 1).

**Table 2:** ORs for associations between different forms of autonomy indicators, relationship inequality, and IPV for married women: 2007 Bangladesh Demographic Health Survey (n = 4,467).

Measure	Any physical and/or sexual IPV			
	COR (99% CI)	*P* Value	AOR (99% CI)	*P* Value§
*Model 1*				
**Overall autonomy index**				
Low	1.00		1.00	
Medium	0.91 (0.71–1.17)	0.345	0.78 (0.59–1.04)	0.026
High	0.62 (0.49–0.78)	**<0.001**	0.48 (0.37–0.61)	**<0.001**
*Model 2*				
**Economic power decision index**				
Low	1.00		1.00	
Medium	1.17 (0.87–1.57)	0.173	0.76 (0.54–1.07)	0.041
High	0.29 (0.22–0.38)	**<0.001**	0.12 (0.08–0.17)	**<0.001**
**Familial health care and family planning decision index**				
Low	1.00		1.00	
Medium	1.00 (0.77–1.31)	0.972	1.30 (0.96–1.77)	0.027
High	0.82 (0.60–1.11)	0.092	1.22 (085–1.77)	0.154
**Index for freedom of movement**				
Low	1.00		1.00	
Medium	1.16 (0.84–1.62)	0.231	1.40 (0.96–2.04)	0.020
High	1.40 (1.02–1.91)	**0.005**	1.94 (1.33–2.85)	**<0.001**
**Index of women's attitudes toward partner's violence**				
Low	1.00		1.00	
Medium	0.77 (0.54–1.09)	0.051	0.76 (0.50–1.14)	0.082
High	0.58 (0.45–0.74)	**<0.001**	0.61 (0.47–0.83)	**<0.001**
*Model 3*				
**Educational inequality**				
Both respondent and her partner were literate	1.00		1.00	
Only husband literate	1.67 (1.20–2.32)	**<0.001**	1.28 (0.90–1.82)	0.073
Only respondent literate	2.14 (1.61–2.83)	**<0.001**	1.73 (1.28–2.34)	**<0.001**
Both respondent and her husband were illiterate	2.21 (1.70–2.87)	**<0.001**	1.59 (1.19–2.13)	**<0.001**
**Occupational inequality**				
Both respondent and her husband had a job	1.00		1.00	
Only husband had a job	0.61 (0.49–0.76)	**<0.001**	0.56 (0.30–0.89)	**0.003**
Only the respondent had a job	1.32 (1.05–2.68)	0.049	1.69 (1.35–2.13)	**0.006**
Both respondent and her husband did not have any job	0.58 (0.30–1.11)	0.031	0.64 (0.26–1.56)	0.197
**Inequality in spousal age at marriage** †				
Both husband and wife married at legal age	1.00		1.00	
Husband or wife married at illegal age	1.93 (1.45–2.57)	<**0.001**	1.38 (1.01–1.89)	0.008
Both husband and wife married at illegal age	2.82 (2.00–3.96)	**<0.001**	1.87 (1.28–2.74)	**<0.001**

Abbreviations: AOR, adjusted odds ratio; CI, confidence interval; IPV, intimate partner violence; COR, crude odds ratio.

† Legal age is 21 years for men and 18 years for women.

§ Using Bonferroni correction for multiple tests the adjusted *P* value is 0.00625 for significance.

Model 1: adjusted for women age, age at marriage, education, husband's education, place, religion, respondent's current occupation status, parity, household member size, frequency of mass media exposure, marital duration, and wealth index.

Model 2: adjusted for women age, age at marriage, education, husband's education, place, religion, respondent's current occupation status, parity, household member size, frequency of mass media exposure, marital duration, wealth index, economic power decision index, familial health care and family planning decision index, index for freedom of movement, and index of women's attitudes toward partner's violence.

Model 3: adjusted for women age, place, religion, parity, household member size, frequency of mass media exposure, marital duration, wealth index, overall autonomy index, educational inequality, occupational inequality, and inequality in spousal age at marriage.

### Crude and Multivariate Analyses

In both the unadjusted (crude odds ratio [COR], 0.62; 99% confidence interval [CI], 0.49–0.78) and adjusted (adjusted odds ratio [AOR], 0.48; 99% CI, 0.37–0.61) models, women who had high overall autonomy were associated with a lower risk of experiencing physical and/or sexual IPV (Table 2). In the unadjusted model, relative to women who had lower economic-decision-making autonomy, women who had high economic-decision-making autonomy had a 0.29 times lower risk of experiencing IPV. After adjustments for socio-demographic variables, high economic-decision-making autonomy (AOR, 0.12; 99% CI, 0.08–0.17; Table 2) was still found to be associated with a lower risk of experiencing IPV.

In the unadjusted model, women who had higher level of non-supportive attitudes toward wife beating or raping were 0.58 times less likely to report having experienced IPV, compared with women who had low level of non-supportive attitudes. After adjustments for confounding factors, a high level of non-supportive attitudes toward wife beating or raping (AOR, 0.61; 99% CI, 0.47–0.83; Table 2) was still found to be associated with a lower risk of IPV. In both the unadjusted and adjusted models, women who had high freedom of movement also were at increased risk of experiencing IPV.

In the unadjusted model, compared with those respondents and husbands who were literate, the remaining categories of literacy discrepancy (illiterate women whose husbands were literate, literate respondents with illiterate husbands, and illiteracy for both spouses) had an increased risk of IPV (Table 2). Adjustments for socio-demographic variables attenuated the strengths of the associations, but they remained significant when only the respondent was literate or when both spouses were illiterate.

In the adjusted model, the risk of IPV was higher when only women were employed (AOR, 1.69; 99% CI, 1.35–2.13), whereas the risk was lower when only the husbands were employed (AOR, 0.56; 99% CI, 0.30–0.89; Table 2). In adjusted model, when both of the spouses were married at an illegal age, the risk of IPV was higher than when both the spouses were married at a legal age.

## Discussion

To the best of our knowledge, the present study is the first to utilize a large nationally representative sample of married Bangladeshi women to examine the relationship between IPV and multidimensional aspects of gender inequity. The present findings indicated that large numbers of married Bangladeshi women experience IPV, with this violence occurring in more than 1 out of 2 such households. This extremely high lifetime prevalence rate is consistent with other previous studies in Bangladesh, including small-scale studies [49]–[50] and a WHO multi-country study [8], and confirms that IPV is a shockingly prevalent problem in this impoverished South Asian nation.

The present findings provide evidence of an association between a high level of women's overall autonomy, particularly a high level of economic-decision-making autonomy and a high level of non-supportive attitudes towards wife beating or raping, and a low risk of experiencing physical and/or sexual IPV among married Bangladeshi women. These findings are an important extension of previous work demonstrating an association between a low level of women's economic-decision-making autonomy [20]–[21] and high agreement with the justification of wife beating or raping and the risk of IPV in developing countries [35], [51]. However, some previous studies have documented negative associations between economic-decision-making autonomy and the risk of IPV [22]–[25].

There are three possible explanations for the discrepancy between the findings of the present study and some of the previous studies that did not find a positive association between economic-decision-making autonomy and the risk of IPV. First, the present study utilized a much larger sample size to examine this relationship than the previous studies, and thus had a sufficient power to detect an association. Second, the discrepancy could be due to methodological issues in constructing the autonomy-related variables. For example, studies in Haiti and Peru [23]–[24] measured economic-decision-making autonomy in terms of female-dominated decision-making power (decision made by the female only), rather than joint decision-making. Third, the discrepancy could be due to a context-specific phenomenon. For example, studies in Bangladesh and Brazil [22], [25] examined these issues in some selected highly culturally conservative rural and urban areas that were characterized by more rigid norms concerning women's roles and status. Our results, therefore, indicate that women's autonomy, particular economic-decision-making autonomy, and women's attitudes toward partner's violence may need to be considered as important socio-cultural determinants for reducing the risk of IPV among women in Bangladesh.

The association between economic-decision-making autonomy and a lower risk of experiencing IPV can be explained by the fact that a woman's control over economic resources may enhance her ability to exercise choice. The obvious benefit is that having control over resources may give her the ability to weigh the costs and benefits of alternative uses of resources, so that they may be employed in the most efficient manner [52]. The more control a woman has over her own time and household income, the more likely that she may have an increased capacity to challenge the acceptability of partner violence, increased expectations of receiving better treatment from her partner, and increased social support through the mobilization of new and existing community groups [53].

The mechanism underpinning the association between a woman's high agreement with partner violence justification and the risk of experiencing IPV can be explained by the high correlation between economic decision-making autonomy and a woman's low agreement with the justification for wife beating or raping. A previous study has shown that empowered women do not agree with any justification for wife beating [51]. Social norms or attitudes that condone or excuse IPV may place women at a greater risk of becoming victims. Women who strongly disagree with the justification of wife beating or raping have a lower chance of occupying a position of inferiority relative to men, which probably makes them less susceptible to IPV. Further work needs to be done to determine, what types of program inputs may alter beliefs and attitudes, and what types of programs may help to empower individuals to work against harmful social norms.

Women who have high freedom of movement autonomy may have more gender-egalitarian beliefs and therefore may be less susceptible to IPV. However, our findings indicated that, high freedom of movement autonomy increased the risk of experiencing IPV. This finding agrees with previously obtained results [15], [54]–[55]. The mechanism underpinning this association can be explained by the high correlation between a woman's employment status and freedom of movement autonomy. This correlation is likely a reflection of the greater prevalence of conventional attitudes toward gender roles among Bangladeshi men and may be a response to a man's feeling of powerlessness.

The present findings also revealed that the education of both men and women led to reductions in IPV. However, an educational gap between a wife and her husband, such as when only the husband was literate or only the wife was literate, actually increased the risk of IPV. These findings suggested the presence of a male backlash: men feel threatened in their traditional masculine gender roles by increases in women's status, especially once the women's achievements trump the achievements of their husbands [56]. The backlash phenomenon has also been observed in other developing countries, such as Albania [57], Peru [24], and India [15]. Therefore, it appears that although the effects of education are largely positive, the story is much more complex than can initially be expected and needs further investigation. It appears that educational interventions do not have a purely beneficial effect if either men or women are the only ones receiving the education. This circumstance suggests the need for more directed educational interventions for both men and women.

Notably, women's occupation status cannot protect them from IPV; the risk of IPV increased when only women were employed, whereas the risk decreased when only the husbands were employed. These findings are once again consistent with the male backlash theory, which predicts that the economic independence of women could actually increase the risk of IPV. Marital relationships are governed by socially and culturally prescribed gender roles. To the extent that women's economic independence challenges socially sanctioned gender roles, women can be subject to IPV because the challenged man might try to reinstate his authority over his wife by inflicting violence on her [58]. In this approach, women's employment, for example, does not merely provide an access to financial resources, but also serves as a symbol that represents the status of men and women within the households. The backlash phenomenon on the effect of the economic independence of women on the risk of IPV has also been observed in other developed [58] and developing [39], [59] countries. Therefore, promoting the empowerment of women in households without the support of men may place women at an increased risk of IPV.

Another new important finding is that when both of the spouses were married at an illegal age, the risk of IPV was higher than that when both of the spouses were married at a legal age. Early marriage may perpetuate an unequal society, increasing female vulnerability, powerlessness, and assetlessness, as well as restricting personal, educational, and psychological development and having hazardous health effects [60]. Moreover, couple**s** who marry at a very young age are likely to live in a dependent situation, being under the care of the**ir** parents, which can cause numerou**s** adjustment problems [61]. A recent study in India found that early marriage was associated with a low involvement in the decision to marry and fewer interactions with one's spouse [61]. All these factors can lead to an elevated risk of IPV. Therefore, effective interventions should be performed to reduce the risk of IPV by reducing the prevalence of child marriage.

The present findings should be interpreted in light of some limitations. First, our analyses were cross-sectional; therefore, we are unable to determine the exact temporal relationship between gender inequity and IPV within the limited timeframe. Longitudinal research on the relationship between gender inequity and IPV is needed to address this issue. Second, this study relied on women's reports of their lifetime experience with IPV, and these reports may have been subjected to recall bias. However to minimize recall bias, different modes of data collection were used, e.g., face-face reporting, confidential self-reports, and the use of memory jogging techniques to aid the recall of events and behavior. Moreover, the rate of lifetime IPV was similar to that reported in a WHO multi-country study on women's health and domestic violence utilizing data from selected urban and rural areas of Bangladesh, where a modified version of the CTS-2 was used [8]. Therefore, we expect that this concern might not be a problem in this particular study. Third, because our selection of variables was constrained by the preexisting BDHS data, we were unable to include additional, potentially important variables.

Finally, the possibility of underreporting must be considered: since IPV is a sensitive and often stigmatized subject, women may be reluctant to reveal their abuse status. However, the personal interview method used in this study is widely used for this type of research [62]–[63]. The BDHS (2007) stated that much care and preparation went into the design and execution of the interviews to create a safe atmosphere in which the respondents would feel comfortable discussing this issue, suggesting that the underreporting or overexploiting of IPV may not be appreciable in this study [27]. Despite these limitations, the results have provided important information that could serve as a basis to reducing the risk of IPV among married Bangladeshi women.

## Conclusions

In conclusion, dimensions of gender inequities were significant predictors of experiencing IPV among married women in Bangladesh. When developing interventions aimed at reducing IPV, gender inequities needs to be considered as important socio-cultural determinates. These findings may also be relevant in other settings where gender inequities persist. Further investigation of the causal link between multidimensional aspects of gender inequity and IPV will be critical to developing interventions to reduce the risk of IPV, and should be considered a public health research priority.

The present findings highlight the need for strategies, programs, and policies that aim to improve women's access to, and notably their control over, financial resources through different types of empowerment programs. Examples of economic interventions, such as micro-credit program, economic livelihoods, and conditional cash transfers (CCTs), have the potential to enhance decision-making abilities and even to reduce IPV [64]. Governments should implement laws to provide more equitable access to assets and services, including land, water, and technology, so as to improve women's economic status and thus strengthen their position. However, much remains to be done in the area of micro-financing, land acquisition and other asset ownership by women to improve their economic situation. There is also a need to identify weaknesses in existing laws to prevent IPV and to abolish child marriage. Families and communities should be educated regarding the importance of a woman's autonomy in marriage decisions. Equally important are efforts to raise awareness among women about their rights and to enhance their ability to challenge existing gender norms through community-based campaigns.
