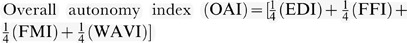# Correction: Does Gender Inequity Increase the Risk of Intimate Partner Violence among Women? Evidence from a National Bangladeshi Sample

**DOI:** 10.1371/journal.pone.0091448

**Published:** 2014-02-28

**Authors:** The PLOS ONE Staff

In the Results section under “Statistical Analyses,” the definition for the Overall Autonomy Index is incorrect. The correct expression is:

Overall autonomy index